# Proteinaceous Microsphere-Based Water-in-Oil Pickering Emulsions for Preservation of Chlorella Cells

**DOI:** 10.3390/polym16050647

**Published:** 2024-02-28

**Authors:** Lin Qi, Teng Hang, Weijie Jiang, Sinong Li, Hui Zhang, Xiang Liang, Le Lei, Qiangqiang Bi, Hang Jiang, Yunxing Li

**Affiliations:** Key Laboratory of Synthetic and Biological Colloids, Ministry of Education & School of Chemical and Material Engineering, Jiangnan University, Wuxi 214122, China

**Keywords:** microalgae, Pickering emulsion, zein, Chlorella cells, high-internal-phase emulsions

## Abstract

Microalgae are highly regarded as ideal materials for the creation of liquid biofuels and have substantial potential for growth and utilization. However, traditional storage and culture methods for microalgae are plagued by challenges such as uncontrolled growth, bacterial contamination, and self-shading among algae. These issues severely impede the photosynthetic process and the efficient extraction of biomass energy. This study tackles these problems by utilizing magnetic hydrophobic protein particles to stabilize water-in-oil Pickering emulsions. This allows for the micro-compartment storage and magnetic transfer of algae. Additionally, the successful encapsulation of Chlorella cells in high-internal-phase water-in-oil Pickering emulsions effectively mitigates the settling problem of Chlorella cells in the liquid phase, thereby enabling the potential use of Pickering emulsions for the confined cultivation of microalgae.

## 1. Introduction

Biomass energy is a renewable energy source that generates less environmental pollution. It is increasingly being recognized as a potential substitute for traditional fossil fuels. Among the various types of biomass, algae offer several advantages, including high biomass production, short growth cycles, easy cultivation, and high lipid content [1,2,3,4]. Oil-producing algae, such as Chlorella vulgaris and Tribonema constrictum, stand out due to their high fat content and promising prospects for further development [5,6,7,8,9]. Compared to other bioenergy crops, algae exhibit faster growth rates and possess higher energy content per unit of biomass.

However, due to the characteristic of algae being prone to rapid reproduction, it is difficult to confine their cultivation within specific areas in natural water bodies [10]. The excessive proliferation of algae outside the designated zones can lead to eutrophication, resulting in water pollution, foul odors, and ecological damage [11]. Moreover, the bacterial contamination of microalgae, insufficient carbon dioxide concentrations in water, and self-shading caused by algal sedimentation can have a substantial impact on algal productivity [12,13]. Consequently, the development of a method for the localized and isolated storage and cultivation of algae is of great significance in enhancing algae cultivation’s efficiency and facilitating bio-extraction.

It is an effective method to isolate and protect microorganisms by using emulsion droplets. Since Devay et al. [14] proposed a method of separating and observing microorganisms with the help of droplets in 1954, the culture and preservation of microorganisms through droplets has been widely applied. A droplet culture system is a tiny emulsion system (usually w/o type) formed by the shear mixing of two incompatible liquids, and each droplet serves as a microculture vessel for the culture of microorganisms. The most obvious advantage of encapsulating and culturing microorganisms in droplets is the micro-compartment [15,16]. Microbial cells are randomly confined to droplet micro-compartments, which can increase microbial diffusion and the transport of nutrients, as well as reduce product inhibition. Moreover, the presence of the external phase can prevent the contact of the microorganisms with the external environment, greatly reducing the possibility of contamination. For example, Leong et al. [17] prepared water-in-oil emulsions using special fluorinated surfactants with the help of a microfluidic device, demonstrating that biocompatible fluorinated surfactant-stabilized emulsions can be used for long-term cell culture. However, surfactant-stabilized emulsion droplets are not completely closed microchambers, which can allow small molecules to pass through, resulting in cross-infection between droplets, which is not conducive to the parallel culture of multiple droplets [18].

Colloidal particles adsorbed at the stable water/oil interface are commonly referred to as Pickering emulsions [19,20,21,22]. Compared to conventional surfactant-stabilized emulsions, the nearly irreversible adsorption of particles at the liquid/liquid interface imparts exceptional stability to Pickering emulsions, making them highly suitable for the protection and delivery of active ingredients, as well as for the preparation of microcapsules and functional materials that exhibit excellent mechanical strength [23,24,25,26,27,28]. Meanwhile, the adsorption of colloidal particles at the interface will form a rigid barrier, which can alleviate the pollution of microbial molecules. Additionally, the surface modifiability and functionalization of colloidal particles provide the ability to selectively control the emulsion types (o/w, w/o, multiple emulsions) and morphologies, thereby enabling the design and fabrication of environmentally responsive Pickering emulsions that respond to stimuli such as pH, temperature, light, and magnetic fields [29,30,31,32,33,34,35,36]. Liu et al. [37] constructed water-in-water (w/w) Pickering emulsions for the microchamber culture of Lactobacillus helveticus, demonstrating that a w/w Pickering emulsion provides a new approach and a great platform for the culture of probiotics. Song et al. [38] used natural magnetotactic bacteria as nanoscale magnetic stirring bars, encapsulated in each microdroplet of the Pickering emulsion, and stirred the solution under the action of an external magnet to significantly improve the catalytic efficiency. This strategy will lead to further innovations in cell culture and application in Pickering emulsions. Hence, Pickering emulsions are believed to serve as ideal micro-compartments for the preservation and growth of microorganisms such as algae, with promising expansion for further innovative applications [21,39,40,41].

Recently, with the promotion of sustainable development and green chemistry concepts, researchers have shifted their research focus to Pickering emulsions stabilized by bio-based or natural particles [42,43,44,45,46]. Biopolymer-based colloidal particles such as starch, cellulose, and proteins have been widely discovered and developed [47,48,49,50,51,52,53]. These biomolecule-based colloidal particles have excellent biocompatibility, natural safety, and sustainability, leading to their successful application in the preparation of food-grade Pickering emulsions, the development of vaccine adjuvants, and drug delivery systems. Similarly, the cultivation of microorganisms, such as microalgae, also requires non-toxic and biocompatible particulate stabilizers [54,55,56]. Furthermore, microalgae need to be stored and grown in the aqueous phase; hence, they must be encapsulated in the dispersed phase of the emulsion, which requires the colloidal particles to be able to stabilize w/o emulsions. However, the hydrophilic nature of biopolymers limits the stabilization of w/o-type Pickering emulsions by their derived colloidal particles.

In our previous study, hydrophobic silica nanoparticles were utilized to stabilize a double emulsion template, followed by the formation of hydrophobic proteinaceous microspheres via the anti-solvent precipitation method, where zein protein served as the skeleton material, and a preliminary assessment of their ability to stabilize w/o Pickering emulsions was conducted [57,58,59]. Herein, using Chlorella cells as the representative microalgae, a water-in-oil (w/o) Pickering emulsion containing dispersed Chlorella cells was prepared. By incorporating Fe_3_O_4_ nanoparticles into the proteinaceous microsphere framework, the resulting emulsion exhibited magnetic responsiveness, enabling the controlled transfer of Chlorella cells. Additionally, increasing the water-to-oil ratio to 3:1 resulted in a closely packed arrangement of w/o droplets (high-internal-phase Pickering emulsions), effectively reducing the issue of sedimentation in the microalgae suspension. Through confinement and isolated storage provided by the w/o Pickering emulsion, it is anticipated that the explosive growth of microalgae, bacterial contamination, and self-shading among algae can be further controlled, thus improving the efficiency of the extraction of microalgae biomass energy.

## 2. Materials and Methods

### 2.1. Materials

Zein (#Z3625) was purchased from Sigma-Aldrich (From St. Louis, MO, USA). Fumed silica nanoparticles (R974) were purchased from Evonik (Located in Essen, Germany). Fe_3_O_4_ nanoparticles, fluorescein isothiocyanate (FITC) and dodecane were purchased from Macklin (From Shanghai City, China). Perylene was purchased from Aladdin (Situated in Shanghai City, China). n-hexane (>97%) and ethanol (>99.7%) were purchased from Sinopharm (From Beijing City, China). A mixture of caprylic/capric triglycerides (GTCC) was purchased from Chou Qin Biotechnology (Located in Rizhao City, China). Chlorella cells were purchased from Futian Bio (Situated in Ningbo City, China). Deionized water was used in all experiments.

### 2.2. Fabrication of Hydrophobic Proteinaceous Microspheres

The fabrication of hydrophobic proteinaceous microspheres referred to our published work [60]. Hydrophobic proteinaceous microspheres were prepared via the Pickering emulsion template method. First, a 70% (*v*/*v*) ethanol aqueous solution of 20% (w/v) zein protein was prepared as the water phase. At the same time, FITC was used for fluorescent labeling. Then, nano-silica R974 as the emulsifier was dispersed in GTCC as the oil phase. All the above solutions were completely ultrasonically dispersed. The oil-in-aqueous-ethanol-in-oil Pickering emulsion was prepared by high-speed homogenizing emulsification for 2 min after mixing with a water-to-oil ratio of 1:5. Then, the ethanol in the emulsion templates was removed for the precipitation of the zein protein via reduced-pressure rotary evaporation. After solvent removal, the microspheres were collected by high-speed centrifugation, purified and washed with n-hexane at least three times, and finally dried in a vacuum drying oven at 50 °C for 12 h to obtain the fluorescent hydrophobic proteinaceous microsphere powder. For the fabrication of magnetic proteinaceous microspheres, 0.5% (w/v) Fe_3_O_4_ nanoparticles were primarily dispersed with zein solution before emulsification, and the other preparation processes were identical.

### 2.3. Preparation of Water-in-Oil Pickering Emulsions and Encapsulation of Chlorella Cells

The w/o Pickering emulsion was prepared with hydrophobic proteinaceous microspheres with a particle concentration of 0.5~5% (w/v) as the emulsifier and dodecane as the oil phase through a simple vortex. In order to investigate the effect of the microsphere concentration on the emulsion formation and droplet size, the ratio of water to oil was 1:3. The use of perylene-labeled oil phases allowed a clearer view of the emulsion type. In the encapsulation experiment with Chlorella cells, the Chlorella cell suspension was diluted at different times (0, 2, 5, 10) as the water phase, and dodecane was used as the oil phase (1% (w/v) microsphere concentration, water/oil ratio 1:3). After hand-shaking, a w/o Pickering emulsion was formed, and Chlorella cells were encapsulated in the droplet. For the preparation of a high-internal-phase Pickering emulsion encapsulating Chlorella cells, the water-to-oil ratio of 3:1 was used, and the other preparation conditions were exactly the same as in the above-mentioned Pickering emulsion preparation.

### 2.4. Characterizations

The morphology of the hydrophobic proteinaceous microspheres was observed by a scanning electron microscope (S-4800, Hitachi Ltd., located in Tokyo, Japan) at a voltage of 3 kV. A video optical contact angle measuring instrument (OCA15EC, Dataphysics, located in Stuttgart, Germany) was used to record the wettability of the hydrophobic proteinaceous microspheres. The Pickering emulsion droplets containing Chlorella cells stabilized by hydrophobic proteinaceous microspheres were observed with an optical microscope (Nikon Ni-U, located in Tokyo, Japan). A confocal laser scanning microscope (CLSM) (Nikon AX equipped with Eclipse Ti2 body, situated in Tokyo, Japan) was used to observe the droplets, the type, the interface structure of the Pickering emulsion, and the state of the Chlorella cells inside the droplet. The excited wavelengths for the Chlorella cells, perylene, and fluorescein isothiocyanate (FITC) were 405 nm, 405 nm, and 488 nm, respectively.

## 3. Results and Discussion

### 3.1. Hydrophobic Proteinaceous Microspheres

**Figure 1:**
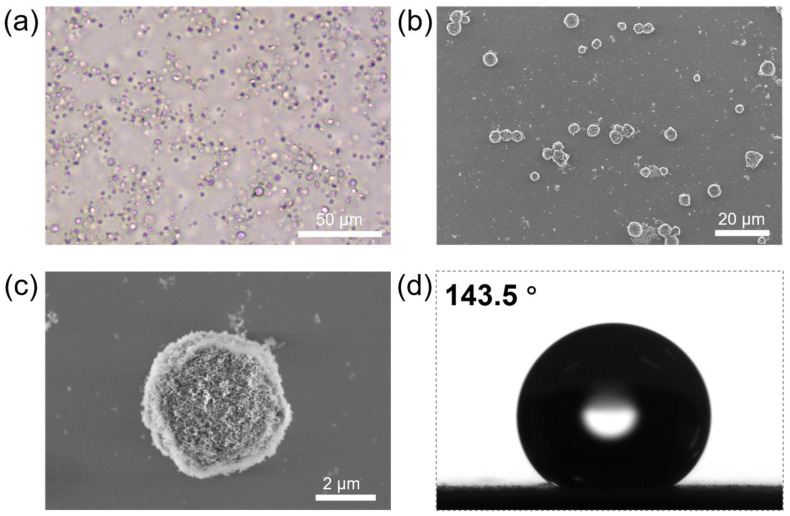
(**a**) Optical microscopic image of the oil-in-aqueous-ethanol-in-oil Pickering emulsion template for the preparation of hydrophobic proteinaceous microspheres. (**b**) SEM image of the fabricated proteinaceous microspheres. (**c**) SEM image illustrating the surface morphology of the proteinaceous microsphere. (**d**) Air/water contact angle of hydrophobic proteinaceous microspheres.

Fumed silica nanoparticles R974 serve as excellent particulate stabilizers for water-in-oil Pickering emulsions [28,61]. Based on our previous research, when mixing an ethanolic solution of zein protein with a GTCC dispersion of R974, it is possible to form an oil-in-aqueous-ethanol-in-oil Pickering double emulsion after high-speed homogeneous shear (Figure 1a) [60]. In this double emulsion template, the outermost liquid/liquid interface is adsorbed and stabilized by the silica nanoparticles, while the intermediate phase consists of the ethanol solution of zein protein. Due to the excellent stability of Pickering emulsions, the integrality of the emulsions can be well maintained even after the ethanol is removed through reduced-pressure rotary evaporation. Additionally, the anti-solvent precipitation of the zein protein results in the solidification of the intermediate phase into a proteinaceous scaffold, ultimately forming proteinaceous microspheres (Figure 1b). The SEM observation reveals that the fabricated proteinaceous microspheres maintain great sphericity even after drying, with a size around 5 μm. Moreover, the surfaces of the proteinaceous microspheres are successfully coated with hydrophobic silica nanoparticles (R974) (Figure 1c); we therefore measured the air/water contact angle of the hydrophobic proteinaceous microspheres and found that they were exceptionally hydrophobic, with a contact angle of up to 143.5°, thereby providing favorable conditions for the subsequent preparation of water-in-oil Pickering emulsions (Figure 1d).

### 3.2. Water-in-Oil Pickering Emulsions Stabilized by Proteinaceous Microspheres

**Figure 2:**
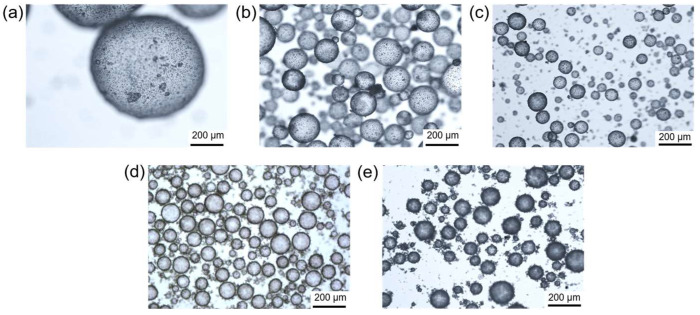
Optical microscopic images of the water-in-oil Pickering emulsions stabilized by proteinaceous microspheres with concentrations of 0.5% (**a**), 1% (**b**), 2% (**c**), 3% (**d**), and 5% (**e**), respectively. All concentrations are mass concentration (w/v).

The Pickering emulsion templating method is a green, mild, and effective approach to regulating the hydrophobicity of proteinaceous particles. Hydrophobic silica nanoparticles are fixed on the surfaces of zein microspheres to improve their hydrophobicity for the stabilization of water-in-oil Pickering emulsions. Figure 2 shows the influence of different concentrations of protein microspheres on the emulsion morphology. The study found that stable water-in-oil Pickering emulsions could still be prepared even at a microsphere concentration of 0.5% (w/v), although the droplet size was relatively large (Figure 2a). Additionally, the adsorption of proteinaceous microspheres on the droplet interface is clearly visible, which helps to reduce the water/oil interfacial energy. Further increasing the microsphere concentration to 1–5% (w/v) results in a significant decrease in droplet size, as shown in Figure 2b–e. However, we can easily find that when the particle concentration reaches 2% (w/v) (Figure 2c), continuing to increase the particle concentration to 3% (w/v) and 5% (w/v) (Figure 2d,e), the size of the emulsion droplets does not decrease significantly. This is because, for most Pickering emulsion systems, it is obvious that an increase in particle concentration can reduce the size of the droplets formed. However, when the particle concentration reaches a certain extent, the droplet size does not change with the increase in particle concentration, and the excess particles tend to disperse in the continuous phase rather than adsorb at the droplet interface. These results indicate that proteinaceous microspheres covered with hydrophobic silica can effectively stabilize water-in-oil Pickering emulsions, providing a potential method for the encapsulation of microalgae or other microorganisms in the internal aqueous phase.

### 3.3. Preservation of Chlorella Cells in Emulsion Droplets

**Figure 3:**
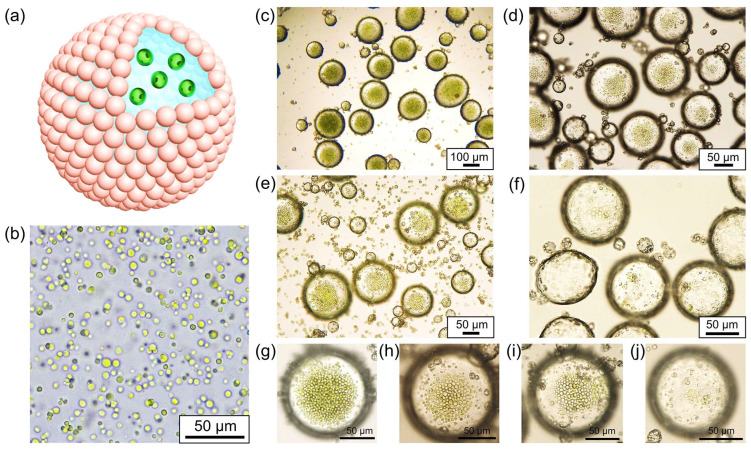
(**a**) Schematic illustration of water-in-oil Pickering emulsion for preservation of Chlorella cells; (**b**) optical microscopic image of the suspension of Chlorella cells; (**c**–**f**) optical microscopic images of the water-in-oil Pickering emulsions for preservation of Chlorella cells with dilution factor of 0, 2, 5, and 10, respectively; (**g**–**j**) magnified optical microscopic images of the emulsion droplets according to the samples in (**c**–**f**), respectively.

Chlorella, belonging to the Chlorophyta phylum, is a unicellular, spherical freshwater alga with cell diameters ranging from 3 to 8 μm. It is renowned for its efficient photosynthetic capabilities, harnessing light energy for photosynthesis [62]. As depicted in Figure 3a, we selected Chlorella as a representative microalga and dispersed it in an aqueous phase to create water-in-oil Pickering emulsions that encased Chlorella cells. According to the literature [63,64], Chlorella cells can not only grow through photoautotrophs but also through organic carbon sources under heterotrophic conditions. Even if there is no light energy input for a period of time, it can survive for a long time by consuming organic matter generated by photosynthesis. This is considered a prerequisite for emulsion encapsulation. Figure 3b displays the relatively uniform size distribution of the Chlorella cells, exhibiting a high degree of sphericity. Due to its high chlorophyll content, Chlorella appears as green spherical particles when observed under a microscope, distinguishing it from proteinaceous microspheres. During the emulsification process, we diluted the original concentrated Chlorella suspension and prepared algae suspensions featuring varied concentrations, which were subsequently employed as the aqueous phase for emulsification. Our findings indicate that the all the Chlorella suspensions of varying concentrations could be effectively emulsified, generating water-in-oil Pickering emulsions wherein the Chlorella cells were distributed within each water droplet (Figure 3c–f). By means of emulsification, the emulsion droplets serve as micro-compartments, preserving the Chlorella cells and effectively isolating them from one another. Furthermore, as the quantity of Chlorella cells in the original suspension decreases, the number of Chlorella cells encapsulated within the emulsion droplets correspondingly diminishes, as illustrated in Figure 3g–j. Consequently, the water-in-oil droplets serve not only as micro-compartments for microalgae storage but also facilitate precise control over the number of microalgae housed within each microchamber.

**Figure 4:**
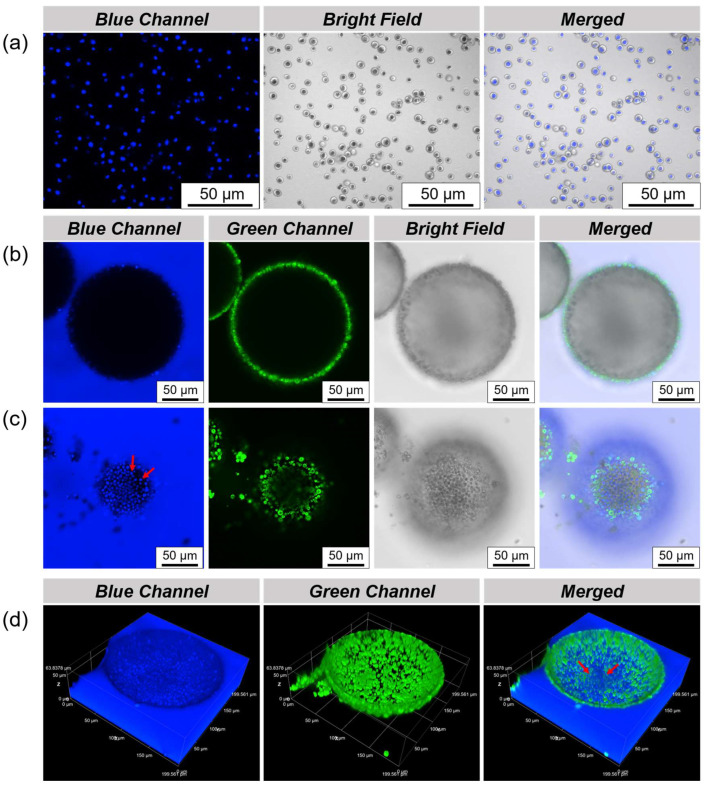
(**a**) CLSM images of the suspension of Chlorella cells; CLSM images of (**b**) cross-sectional focus and (**c**) bottom focus of the water-in-oil Pickering emulsions for the preservation of Chlorella cells, in which the proteinaceous microspheres and oil phase were separately labeled with FITC and perylene; Among them, Chlorella cells are highlighted by red arrows; (**d**) three-dimensional reconstruction images of the droplet preserving Chlorella cells, the red arrow acts as above.

In order to more clearly observe and analyze the state of the Chlorella cells in the droplet microchamber, CLSM was used to characterize it. Chlorella cells can still emit blue fluorescence without the use of fluorescent labels in order to be easily distinguished from the proteinaceous microspheres labeled by FITC, as shown in Figure 4a. After emulsification, Chlorella cells can be encapsulated inside the droplet, using a perylene-labeled oil phase, and the state of the Chlorella cells inside the droplet can be clearly seen by CLSM. It is worth noting that at the cross-sectional focus of the droplet, only the adsorption of proteinaceous microspheres emitting green fluorescence on the interface can be presented, and the presence of Chlorella cells is not observed (Figure 4b). However, from the image of the bottom focus, it can be observed that the Chlorella cells are completely sedimented at the bottom of the droplet, as indicated by the red arrow (Figure 4c). As shown in Figure 4d, by 3D scanning the droplet, we can understand this phenomenon more intuitively and three-dimensionally. The reason that Chlorella cells sediment to the bottom of the droplets is obvious due to the effect of gravity.

### 3.4. Storage Stability of the Pickering Emulsions with Encapsulation of Chlorella Cells

**Figure 5:**
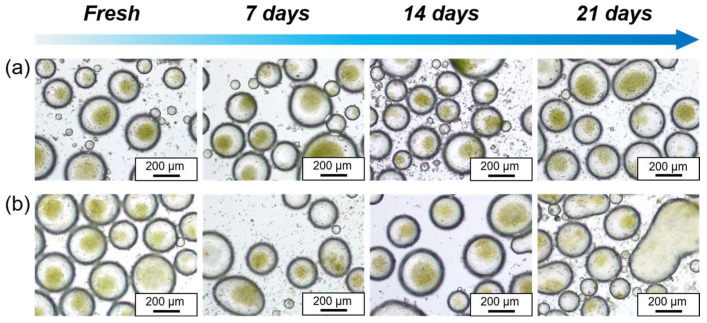
Optical microscopic images of the water-in-oil Pickering emulsions for preservation of Chlorella cells with time, stored at 4 °C (**a**) and 25 °C (**b**), respectively.

Replacing surfactant-based water-in-oil emulsions with particle-stabilized emulsions is an effective way to prevent damage to microalgae caused by surfactants. However, both storage and culture require the droplet to be stable for a longer period of time, thereby preventing the Chlorella cells from leaking out of the droplet micro-compartments, resulting in the loss of this encapsulation protection. Therefore, it is essential to ensure the stability of these microalgae micro-compartments. In this study, we investigated the stability of algae suspensions stored in water-in-oil Pickering emulsions at both refrigerated conditions (4 °C) and room temperature (25 °C). As shown in Figure 5a,b, the water-in-oil Pickering emulsions containing Chlorella cells demonstrated excellent stability during the initial 7 days under both refrigerated and room temperature conditions, and no demulsification or leakage of Chlorella cells was observed and was a minimal change in the droplet size. Nevertheless, on the 14th day, the water-in-oil Pickering emulsions at room temperature began to exhibit signs of instability, with some droplets deforming and coalescing. By the 21st day, significant demulsification and separation occurred when stored at room temperature.

It is noteworthy that the emulsion under refrigerated conditions maintained great stability even after 21 days, showing no apparent demulsification, leakage of microalgae, or significant change in droplet size (Figure 5a). Typically, the original algae suspension is stored under refrigeration to ensure the maximum viability of the microalgae when in use. Therefore, the emulsion compartment system can serve as a novel and stable platform for the long-term storage of microalgae, particularly when refrigerated.

### 3.5. High-Internal-Phase Pickering Emulsions for Preservation of Chlorella Cells

**Figure 6:**
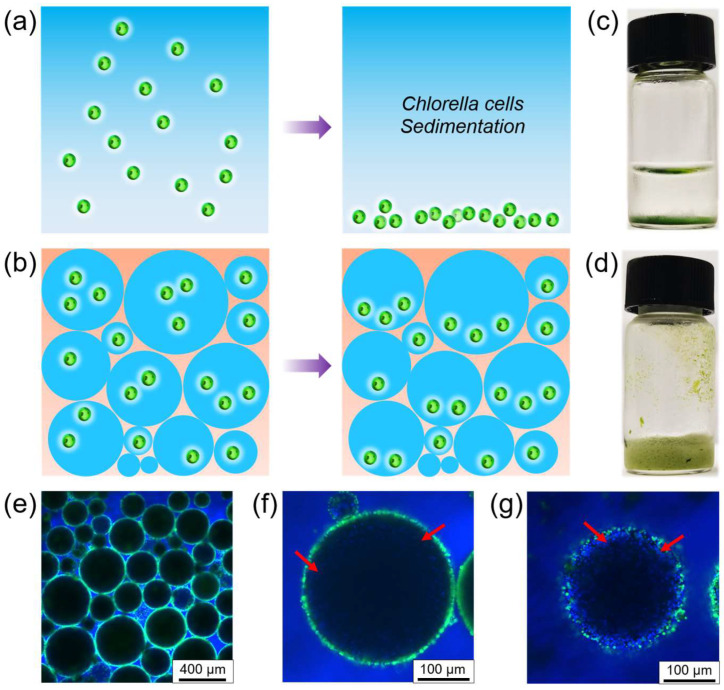
(**a**) Schematic illustration of the sedimentation of Chlorella cells in water; (**b**) schematic representation of the sedimentation of Chlorella cells within the water-in-oil high-internal-phase Pickering emulsion droplets; (**c**) photo of the suspension of Chlorella cells after 4 days; (**d**) photo of the water-in-oil high-internal-phase Pickering emulsion encapsulating Chlorella cells after 4 days; (**e**) CLSM image of the water-in-oil high internal phase Pickering emulsion encapsulating Chlorella cells, in which proteinaceous microspheres and oil phase were separately labeled with FITC and perylene; CLSM images of (**f**) cross-sectional focus and (**g**) bottom focus of a single droplet with high-internal-phase Pickering emulsion. Among them, Chlorella cells are highlighted by red arrows.

Microalgae are easily sedimented at the bottom during storage and culture due to the influence of gravity. This has an impact on the activity and photosynthetic efficiency of microalgae, as illustrated in Figure 6a. Improving the viscosity of the Pickering emulsion so that the droplets are not easily sedimented is an excellent way to solve this problem. Based on this, we increased the water-to-oil ratio to 3:1 to successfully prepare water-in-oil high-internal-phase Pickering emulsions (HIPEs) [65,66], which greatly enhanced the overall viscosity of the emulsion and effectively slowed down the sedimentation of the emulsion droplets. Although the Chlorella cells still sedimented to the bottom of the droplet, the overall state was dispersed, as depicted in Figure 6b. Therefore, we encapsulated Chlorella cells in water-in-oil HIPEs, aiming to minimize the sedimentation of microalgae using this method. Compared to a normal microalgae suspension (Figure 6c), water-in-oil HIPEs used for microalgae encapsulation show no significant sedimentation, even after 4 days of storage (Figure 6d). The emulsion type of HIPEs (Figure 6e) and the successful encapsulation of Chlorella cells (Figure 6f,g) can be demonstrated by CLSM images. This approach holds the potential to provide a new platform and microenvironment for the cultivation and study of microalgae photosynthesis.

### 3.6. Magnetic Responsiveness of the Pickering Emulsions for Storage of Chlorella Cells

**Figure 7:**
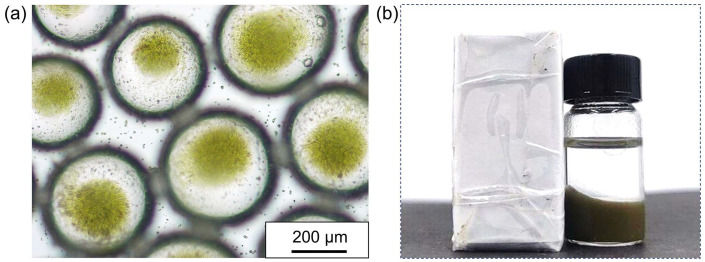
(**a**) Optical microscopic image of the water-in-oil Pickering emulsion stabilized by magnetically responsive proteinaceous microspheres for preservation of the Chlorella cells; (**b**) photo showing the magnetic responsiveness of the water-in-oil Pickering emulsion with the encapsulation of the Chlorella cells.

Another pressing problem after the successful encapsulation of Chlorella cells is how to transfer them to specific sites without breaking the emulsion or damaging the Chlorella cells. As a particle-stabilized emulsion, the properties of Pickering emulsions are largely determined by the properties of the emulsifier particles themselves. The surface modifiability and functionalization of colloidal particles provide the ability to selectively control the morphology of emulsions, enabling the design and manufacture of environmentally sensitive Pickering emulsions that can respond to stimuli such as pH, temperature, light, and magnetic fields. Among them, magnetism is undoubtedly the most classic and lowest-energy response. Therefore, rapid transfer through magnetism is an excellent way to solve this problem. Based on this, introducing Fe_3_O_4_ nanoparticles into proteinaceous microspheres during the particle preparation process can confer magnetic responsiveness to the formed proteinaceous microspheres, thereby achieving the magnetic responsiveness of the as-prepared water-in-oil Pickering emulsions, so as the micro-compartments encapsulating the Chlorella cells can be effectively transported by a magnetic field. In order to demonstrate that magnetically responsive hydrophobic proteinaceous microspheres still have excellent emulsifying abilities, we first used them as an emulsifier to stabilize the w/o Pickering emulsion. It was found that the magnetically responsive proteinaceous microspheres did not affect the preparation of the water-in-oil Pickering emulsions and the encapsulation of the Chlorella cells (Figure 7a). Notably, after the Pickering emulsion became magnetically responsive, the collection and transfer of emulsion droplets could be easily achieved using a regular magnet (Figure 7b). We believe that under the action of an external magnetic field, the droplets containing Chlorella cells can move in a specified direction and can be transferred to a pre-set environment for a period of culture and then demulsified separation. At the same time, we can magnetically recover the proteinaceous microspheres after demulsification, so as to achieve the purpose of recycling. This method holds promise for potential applications in the cultivation of microalgae in the future.

## 4. Conclusions

We prepared hydrophobic proteinaceous microspheres using emulsion templates and embedded magnetic nanoparticles to confer magnetic responsiveness to the proteinaceous microspheres. These proteinaceous microspheres can be used in stabilizing and preparing water-in-oil Pickering emulsions to encapsulate microalgal cells inside droplets. The emulsion system containing microalgae cells can be stable for at least 21 days at 4 °C, serving as a novel microenvironment platform for the preservation of microalgae cells. By increasing the ratio of the internal water phase, the Chlorella cells were encapsulated in a high-internal-phase Pickering emulsion. The potential activities and self-shielding issues caused by algal sedimentation can be expected to be effectively mitigated, which provides great potential for the cultivation and photosynthesis of microalgae in the future. Concurrently, the use of magnetically responsive Pickering emulsions can be taken in consideration to solve the problem of the directional transfer of Chlorella cells without breaking the emulsion or damaging the Chlorella cells.

## Data Availability

Data are contained within the article.
